# Neuroprotective Mechanisms of Resveratrol in Alzheimer's Disease: Role of SIRT1

**DOI:** 10.1155/2018/8152373

**Published:** 2018-10-30

**Authors:** Bruno Alexandre Quadros Gomes, João Paulo Bastos Silva, Camila Fernanda Rodrigues Romeiro, Sávio Monteiro dos Santos, Caroline Azulay Rodrigues, Pricila Rodrigues Gonçalves, Joni Tetsuo Sakai, Paulo Fernando Santos Mendes, Everton Luiz Pompeu Varela, Marta Chagas Monteiro

**Affiliations:** ^1^Neuroscience and Cell Biology Graduate Program, Institute of Biological Sciences, Federal University of Pará, Belém, Pará, Brazil; ^2^Faculty of Pharmacy, Institute of Health Sciences, Federal University of Pará, Belém, Pará, Brazil; ^3^Pharmaceutical Sciences Graduate Program, Institute of Health Sciences, Federal University of Pará, Belém, Pará, Brazil

## Abstract

Alzheimer's disease (AD) is a progressive and neurodegenerative disorder of the cortex and hippocampus, which eventually leads to cognitive impairment. Although the etiology of AD remains unclear, the presence of *β*-amyloid (A*β*) peptides in these learning and memory regions is a hallmark of AD. Therefore, the inhibition of A*β* peptide aggregation has been considered the primary therapeutic strategy for AD treatment. Many studies have shown that resveratrol has antioxidant, anti-inflammatory, and neuroprotective properties and can decrease the toxicity and aggregation of A*β* peptides in the hippocampus of AD patients, promote neurogenesis, and prevent hippocampal damage. In addition, the antioxidant activity of resveratrol plays an important role in neuronal differentiation through the activation of silent information regulator-1 (SIRT1). SIRT1 plays a vital role in the growth and differentiation of neurons and prevents the apoptotic death of these neurons by deacetylating and repressing p53 activity; however, the exact mechanisms remain unclear. Resveratrol also has anti-inflammatory effects as it suppresses M1 microglia activation, which is involved in the initiation of neurodegeneration, and promotes Th2 responses by increasing anti-inflammatory cytokines and SIRT1 expression. This review will focus on the antioxidant and anti-inflammatory neuroprotective effects of resveratrol, specifically on its role in SIRT1 and the association with AD pathophysiology.

## 1. Introduction

Alzheimer's disease (AD) is a neurodegenerative pathology that causes impaired cognitive functioning and memory [1, 2]. Despite the disease being identified over 100 years ago [3], efforts are currently being expended to discover new chemical products (i.e., natural antioxidants) that act at determined points to block the progression of the disease [4, 5]. Resveratrol has been considered as a protector compound for the treatment of neurodegenerative diseases (i.e., AD, Parkinson disease, and amyotrophic lateral sclerosis) that have high levels of oxidative damage due to its antioxidant and anti-inflammatory properties [6]. Moreover, this compound can also modulate different molecular pathways dependent on silent information regulator-1 (SIRT1) in neurodegenerative diseases [6]. However, recent reviews also report other multipathways that are involved in the neuroprotective mechanisms of resveratrol such as inhibition of nuclear factor-*κ*appa B (NF-*κ*B) expression and alteration in the signaling pathways of mitogen-activated protein kinases (P38-MAPK), extracellular signal-regulated kinase 1/2 (ERK1/2) and phosphoinositide 3-kinase (PI3K)/Akt, activation of autophagy, among others [7–10].

Interest in resveratrol has grown recently due to its beneficial effects in several neurological and autoimmune disorders [11, 12]. Resveratrol is a phytoalexin that mainly occurs in grapevine species (*Vitis* sp.) and other fruits, and attention has been drawn to it due to its versatile biological properties, including its antioxidant, anti-inflammatory, and neuroprotective activities [13–15]. In this sense, resveratrol could indirectly activate SIRT1 expression [16] and lead to neuroprotection in AD cases [17]. SIRT1 regulates the activity of several substrates, including p53 and peroxisome proliferator-activated receptor-gamma coactivator 1*α* (PGC-1*α*) [18], which decrease the accumulation of *β*-amyloid (A*β*) and improve mitochondrial dysfunction [19].

Some studies have shown that resveratrol improves the impaired learning and memory in neurodegenerative disease and protects the memory decline in AD through its antioxidant activity [20]. Resveratrol is also effective at preventing blood-brain barrier (BBB) impairment and inhibiting A*β*1–42 from crossing the BBB and accumulating in the hippocampus [21, 22]. The hippocampus is a critical brain component for cognitive and memory functions, is a region that displays ongoing neurogenesis in adulthood, and is a very sensitive area in AD [23–25]. However, a significant reduction in hippocampal neurodegeneration was observed after intracerebroventricular injection of resveratrol in an animal model, which was associated with a decrease in SIRT1 acetylation [26, 27].

Karuppagounder et al. [28] showed that mice treated with resveratrol for 45 days had reduced A*β* toxicity. This suggests that the onset of neurodegeneration may be delayed by dietary chemopreventive agents (i.e., resveratrol) that protect against A*β* formation and oxidative stress [28]. Wang et al. [29] recently showed that resveratrol protected neurons against A*β*1–42-induced disruption of spatial learning, memory, and synaptic plasticity and rescued the reduction of SIRT1 expression in hippocampal rats. Thus, resveratrol is effective at reducing central nervous system (CNS) damage and decreasing the ischemia and toxicity induced by A*β* peptide, showing its potential therapeutic use in neurodegenerative diseases [30].

One of the major neuroprotective mechanisms of resveratrol is the activation of SIRT1 that is expressed in the adult mammalian brain, predominantly in neurons [31]. Activation of SIRT1 by resveratrol prevents A*β*-induced microglial death and contributes to improved cognitive function [32]. Although the major mechanisms of resveratrol are associated with the overexpression of SIRT1, its subsequent neuroprotective effect remains unknown. However, the overexpression of SIRT1 plays an important role in neuronal protection as it regulates reactive oxygen species (ROS), nitric oxide (NO), proinflammatory cytokine production, and A*β* expression in the brains of AD patients [33–36]. This review discusses the neuroprotective effects of resveratrol that are dependent on its action on SIRT1 and its implications in AD.

## 2. Resveratrol Plant Biosynthesis and Pharmacokinetics

Resveratrol (3,5,4′-trihydroxy-trans-stilbene) is a polyphenol plant secondary metabolite that has a phytoalexin role in high plant species. This metabolite is commonly found in grapevines (*Vitis vinifera*), grape juice, and wine [37, 38]. Others food sources, including peanuts, pomegranate, spinach, and bananas, also contain high concentrations of resveratrol [39–43].  Table 1 shows the concentration of resveratrol in some food sources.

**Figure 1:**
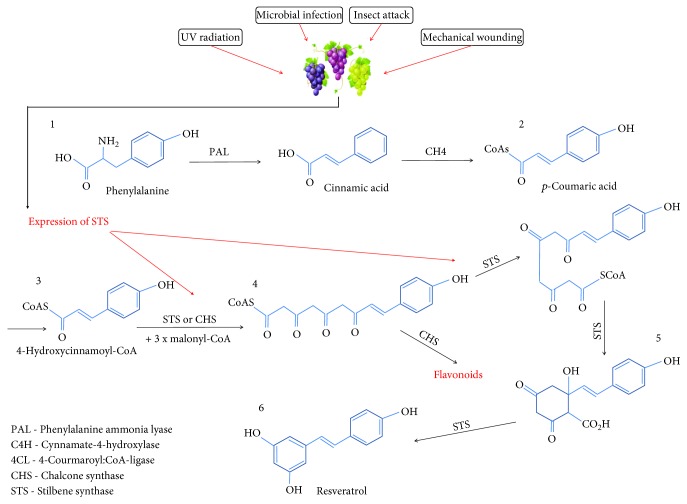
Resveratrol biosynthesis route in high plants.

Resveratrol is synthesized in high plant species using the phenylpropanoid pathway under biotic and abiotic stress conditions (i.e., ultraviolet (UV) light radiation and tissue disruption) and in response to fungal infections (i.e., *V. vinifera* leaves infected by *Plasmopara viticola*) [44–46]. The biosynthesis of resveratrol begins with the generation of 4-coumaroyl-CoA units in the phenylpropanoid pathway [47]. At this point, stilbene synthase (STS) and chalcone synthase (CHS) enzymes promote the chain extension of 4-coumaroyl-CoA via the addition of three malonyl-CoA molecules to generate a polyketide compound (Figure 1). Despite both enzymes using the same substrate, STS possesses substantially more amino acids than CHS (the key enzyme in flavonoid biosynthesis), which explains the difference in the end products formed [48, 49].

The polyketide peptide suffers a fold that promotes the generation of aromatic rings in a Claisen-like reaction catalyzed by STS, which produces an unstable intermediate metabolite called stilbene-2-carboxylic acid [50, 51]. The final steps involve the stepwise reactions that promote the decarboxylation, dehydration, and enolization of stilbene-2-carboxylic acid to yield the resveratrol molecule [52]. Resveratrol can undergo other biochemical reactions to produce new stilbenes, including *ε*-viniferin, t-piceid, t-piceatannol, and t-pterostilbene [53].

Resveratrol is well absorbed but is quickly excreted, mainly by the urinary system [54]. Calliari et al. [55] reported that the pharmacokinetics of resveratrol have been studied in several organs and that its therapeutic effect is mainly dose dependent. After oral consumption, resveratrol is primarily metabolized by phase II enzymes, especially glucuronides and sulfatases, and absorbed in the small gut, predominantly in its glucuronidated form [12, 56]. In addition to the glucuronide metabolite, sulfated products of resveratrol are also commonly found in biological samples [57]; however, only trace amounts of free resveratrol can be detected in plasma [58]. In this regard, Sergides et al. [59] demonstrated higher plasma concentrations of glucuronidated (4083.9 ± 1704.4 ng/ml) and sulfated (1516.0 ± 639.0 ng/ml) resveratrol than its unmetabolized form (71.2 ± 42.4 ng/ml) following the consumption of a single resveratrol (500 mg) tablet in healthy volunteers. Resveratrol is mainly attained by dietary intake; however, there are some concerns regarding its low concentration in food sources and its poor oral bioavailability. This has highlighted the need for strategies that allow biologically active concentrations of resveratrol to reach its target tissues, including the brain [60]. In this regard, Oliveira et al. [12] reported that the major problem of resveratrol treatment was its low bioavailability, with some human studies reporting that even high-dose resveratrol treatment (500 mg/day) produced low plasma concentrations (10–71.2 ng/ml) of this antioxidant.

The description of resveratrol concentrations in the brain is a challenge that remains to be overcome. Frozza et al. [61] reported that intravenous administration of resveratrol reached satisfactory target brain regions, while oral resveratrol treatment was not well absorbed and resulted in reduced stability, increased photosensitivity, and accelerated metabolism, thus making it difficult to reach the brain. Turner et al. [62] showed that resveratrol and its metabolites crossed the human BBB, and these authors detected resveratrol in both the plasma and cerebrospinal fluid, thus showing its effects on the CNS. Preclinical data suggest that the main metabolite found in the rat brain after resveratrol consumption is resveratrol-3-glucuronic acid, which is also the main metabolite found in plasma [63]. To try to overcome the low oral bioavailability, several researchers focused on the microencapsulation technique or on the creation of prodrugs that, after metabolization, will give rise to resveratrol molecules [12, 64, 65]. Studies with new conjugated particles that improve the pharmacokinetics of resveratrol in the brain are of great importance, as the biologically active concentrations observed in *in vitro* experiments are much higher than those achieved after oral consumption are. Frozza et al. [61, 66] demonstrated that resveratrol nanoparticles reached the brain at higher concentrations than free resveratrol, resulting in increased bioavailability and possible neuroprotective effects. Resveratrol is considered a low-toxic substance, as humans have used several resveratrol-containing foods for a long time without related toxic effects. Data also confirm the safety of resveratrol on the basis of preclinical tests and clinical trials [67, 68].

Some studies have reported that resveratrol is an activator of SIRT1 [27, 69], although further evidence shows that resveratrol is not a direct activator of SIRT1 [70], and that its role may be related to the activation of substrates of SIRT1 [71]. The overexpression of SIRT1 results in neuroprotection in AD [17]. SIRT1 inhibits NF-*κ*B signaling by decreasing A*β*-induced toxicity in primary mouse neuronal cultures [32]. SIRT1 may be capable of determining A*β* production by modulating *β*-secretase 1 expression through NF-*κ*B signaling [32].

## 3. Role of SIRT1 in the Pathophysiology of AD

Oxidative stress and the overproduction of ROS are associated with the pathophysiology of neurodegenerative disorders, including AD, and lead to neural membrane injury and memory impairment [72–75]. Brain tissue is more susceptible to oxidative stress due to its high oxygen consumption rate, low regenerative capability, high polyunsaturated fatty acid content, and low concentration of antioxidants [76, 77]. ROS are major neurotoxic factors released by activated microglia and include superoxide radicals (O_2_^·^), hydroxyl radicals (^·^OH), and hydrogen peroxide (H_2_O_2_). These molecules are highly reactive, and their excessive production can induce lipid peroxidation, (deoxyribonucleic acid) DNA fragmentation, and protein oxidation and result in further cell dysfunction and cell death [78]. Therefore, mitochondria that are damaged during oxidative stress can produce ROS that damage proteins, nucleic acids, and polyunsaturated fatty acid membranes and cause lipid peroxidation, a loss of membrane integrity, and increased calcium (Ca^2+^) permeability. ROS also increase the production of A*β* peptides, which induce oxidative stress both *in vitro* and *in vivo* [79]. Thus, a vicious cycle between ROS and A*β* accumulation may accelerate the progression of AD [80]. Studies *in vitro* and *in vivo* have shown that ROS increases A*β* production and induces oxidative stress, thus leading to neuronal apoptosis and accelerating the progression of AD [80–82].

AD is a progressive neurodegenerative disorder of the cortex and hippocampus that eventually leads to cognitive impairment. Although the etiology of AD remains unclear, multiple cellular changes have been implicated, including the production and accumulation of A*β* peptides, tau phosphorylation, oxidative stress, mitochondrial dysfunction, synaptic damage, and biometal dyshomeostasis. The neuroinflammatory response via microglial activation and acetylcholine deficits are also considered to play significant roles in the pathophysiology of AD [83, 84]. The main pathogenic event in AD is the cerebral aggregation of A*β* peptides [85]. A*β* is the major constituent of plaques and is generated from amyloid precursor protein (APP) by the action of *β* and *γ*-secretases [86]. The accumulation of A*β* could initiate a series of downstream neurotoxic events that result in neuronal dysfunction in AD patients [87, 88]. However, oxidative stress is also an important event in the pathogenesis of AD [89], as the generation and accumulation of ROS and reactive nitrogen species can accelerate fibrillization, increase the toxicity of A*β*, and promote neuronal death and neurodegeneration [90–93].

Decreased sirtuin levels, mainly SIRT1 expression levels, were recently correlated with elevated A*β* production and deposition in AD patients [94]. SIRT1 may regulate A*β* metabolism through the modulation of APP processing, and loss of SIRT1 is closely associated with exacerbated A*β* production [95]. However, SIRT1 overexpression decreases A*β* production [95, 96], which may represent an interesting therapeutic approach to block the neurodegeneration and cognitive impairments caused by the disease. SIRT1 is a member of a sirtuin family that utilizes nicotinamide (NAD^+^) as a substrate to catalyze the deacetylation of various substrates [97]. SIRT1 plays an essential role in regulating cellular homeostasis by influencing neuron survival, insulin sensitivity, glucose metabolism, and mitochondrial biogenesis [98, 99]. In the adult brain, SIRT1 was shown to be essential for synaptic plasticity, cognitive functions [100], and the modulation of learning and memory function [101].

**Figure 2:**
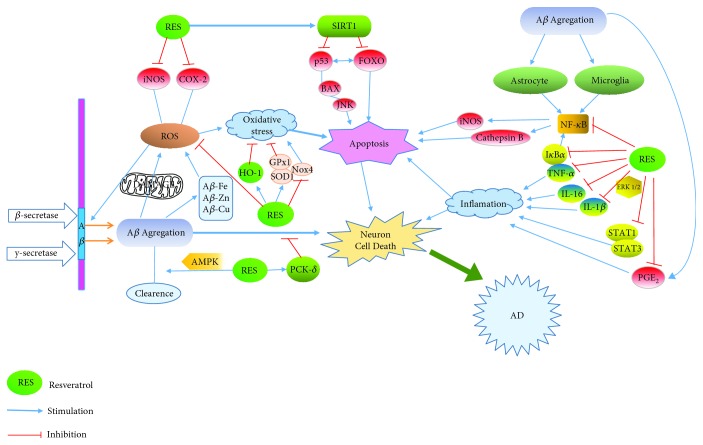
Main cellular routes proposed for the mechanisms of resveratrol in Alzheimer's disease. Modified from Ma et al. [72].

During normal aging, SIRT1 is responsible for the maintenance of neural systems and behavior, including the modulation of synaptic plasticity and memory processes [102]. The absence of SIRT1 expression in hippocampal neurons is correlated with impaired cognitive abilities, including immediate memory, classical conditioning, and spatial learning [100]. SIRT1 can also increase PGC-1*α* activity, which leads to the inhibition of A*β* production and improved mitochondrial dysfunction [19]. SIRT1 can also deacetylate a large number of other substrates, including p53, NF-*κ*B, and Forkhead box O (FOXO), and prevent neuronal apoptosis [103, 104]. Therefore, the pharmacological activation of SIRT1 may represent a promising approach to preventing A*β* deposition and neurodegeneration in AD [105]. Thus inhibiting ROS production may be an important tool for protecting neuronal cells from oxidative damage and a therapeutic strategy in the treatment of neurological disorders [106].  Figure 2 summarizes the pathways by which resveratrol acts on SIRT1 in the pathology of Alzheimer's disease.

### 3.1. Antioxidant Mechanisms of Resveratrol in AD: Role of SIRT1

Oxidative stress induces neuronal damage, modulates intracellular signaling, and leads to neuronal death by apoptosis or necrosis. Therefore, antioxidant products (i.e., resveratrol) are used to protect against neuronal damage in neurodegenerative disorders (i.e., AD) [80]. The antioxidant properties of resveratrol were reported in several studies, which demonstrated that chronic resveratrol treatment reduced the production of malondialdehyde and nitrite and restored glutathione (GSH) levels [107, 108]. Additional antioxidant mechanisms of resveratrol were also described and include SIRT1 activation, A*β* aggregation and toxicity inhibition, metal chelation, and ROS scavenging [106, 108, 109]. These results demonstrate that this compound is an effective therapeutic strategy for AD therapy. Therefore, resveratrol not only plays a role in ROS protection but it can also modulate important glial functions, including glutamate uptake activity, GSH, improved functional recovery, and decreased DNA fragmentation and apoptosis [110–112].

#### 3.1.1. In Vitro Studies

Resveratrol can dysregulate the metal ion balance (i.e., copper, zinc, and iron) and play a key role in neurodegeneration, which is related to cellular function changes and neuronal survival dysfunction [27]. These metal ions are able to bind A*β* and neurofibrillary tangles and promote their aggregation [106, 109], enhance the production of ROS, and contribute to AD pathogenesis. Hou et al. [113] demonstrated the interaction between resveratrol and SIRT1 using molecular dynamics simulation. The authors proposed that resveratrol was responsible for enhancing the binding affinity between SIRT1 and the substrate, thus functioning as a binding stabilizer. Nevertheless, Dasgupta and Milbrandt show that resveratrol is a potent activator of AMP-activated protein kinase (AMPK) function, and resveratrol-mediated AMPK activation was independent of SIRT1 [114]. In addition, in cell lines, resveratrol presented a decrease in the acetylation of PGC-1*α*, possibly due to the activation of AMPK [115]. Thus, showing a dose-dependent effect, resveratrol was able to activate AMPK independently of SIRT1 [116]. However, SIRT1 plays a key role in protecting neurons from the oxidative effects of ROS, NO, and A*β* peptides in the brains of AD subjects [117].

#### 3.1.2. Animal Studies

One neuroprotective property attributed to resveratrol is the suppression of ROS formation through the inhibition of prooxidative genes (i.e., nicotinamide adenine dinucleotide phosphate oxidase) [118]. Huang et al. [119] showed that the neuroprotective activity of resveratrol included the suppression of inducible nitric oxide synthase (iNOS) production, which is involved in A*β*-induced lipid peroxidation and heme oxygenase-1 downregulation, thereby protecting the rats from A*β*-induced neurotoxicity [120]. Moreover, resveratrol induced the expression of various antioxidant enzymes, such as superoxide dismutase (SOD), catalase, thioredoxin, and glutathione peroxidase (GPx) [121, 122]. However, Lee et al. [123] showed that resveratrol possesses chelator-metal ion properties to attenuate the metal imbalance and ROS production [124]. Furthermore, the oral administered of resveratrol in mice lowered the A*β* accumulation in the cortex due to the activation of AMPK signaling by enhancing cytosolic Ca^2+^ levels in neuronal cultures [120, 125].

Other studies also showed the neuroprotective action of resveratrol in animal models; for example, Simão et al. [126] evaluated the response to a 7-day resveratrol treatment (30 mg/kg) on postinduced ischemia in rodent models. Cerebral immunohistochemistry showed reduced activation of astrocytes and microglia in the hippocampus and suppression of the inflammatory response mediated by NF-*κ*B, cyclooxygenase 2 (COX-2), and nitric oxide synthetase (NOS) in hippocampal cells, thus suggesting the anti-inflammatory potential of resveratrol in brain damage. Moreover, Wang et al. [127] suggested that resveratrol (200 mg/kg/day for 8 weeks) could act as an AD-adjuvant therapy after human umbilical cord stem cell transplantation. This occurred due to the increased expression of brain-derived neurotrophic factor precursor (BDNF), neuronal growth factor (NGF), and neurotrophin 3 (NT-3), which are associated with neurogenesis, survival, learning, and memory. Thus, resveratrol positively stimulated these cell-protected factors [128]. The overexpression of these neurotrophic factors is related to the ability of resveratrol to increase the activity of SIRT1 [13]. Similarly, resveratrol also induced an increase of SIRT1 in a mice model [129]. Another study also reported the preventive action of resveratrol in decrease the formation of insoluble A*β* plaques in the hippocampus of rats [21], as the etiology of the disease is associated with an imbalance in A*β* homeostasis. Resveratrol effectively reduced the cleavage activation of APP and promoted peptide clearance [10]; therefore, the authors suggested that resveratrol was efficient at reducing the formation of protein aggregates.

#### 3.1.3. Human Studies

There are currently studies evaluating the effectiveness of resveratrol in AD; for example, a randomized double-blind placebo-controlled study evaluated the effects of resveratrol in 64 AD patients with a mild form of the disease. A resveratrol dose of 500–1000 mg was administered orally to these patients. However, the results demonstrate that resveratrol and its major metabolites able to cross the BBB and cause weight loss and reactions such as nausea and diarrhea. In addition, brain volume loss was greater in the group receiving resveratrol. Conversely, Imamura et al. [130] demonstrated the antioxidant effect of resveratrol on arterial stiffness in patients with type 2 diabetes mellitus (T2DM). In this randomized double-blind placebo-controlled clinical trial, 50 patients were selected: 25 received resveratrol (100 mg/day) and 25 received a placebo for 12 weeks. Supplementation with resveratrol improved several parameters in the T2DM patients and decreased oxidative stress, which was evaluated through metabolites of reactive oxygen. Mansur et al. [131] also conducted a study to evaluate the effects of resveratrol in humans. Slightly overweight elderly individuals were randomly divided into two groups: group one received 250 mg of resveratrol orally twice daily, while group two received a caloric restriction diet (1000 cal/day). SIRT1 concentrations were determined in both groups at the end of the 30-day treatment period. The serum concentration of SIRT1 was increased in both groups; however, this finding was not correlated with a better profile of metabolic markers for atherosclerotic processes.

### 3.2. SIRT1 and Anti-Inflammatory Mechanisms of Resveratrol

Neuroinflammation is an important contributor to the pathogenesis of AD [132]. Various reports show that inflammatory responses occur in the CNS, including the activation of microglia, astrocytes, lymphocytes, and macrophages that trigger numerous proinflammatory mediators and neurotransmitters [133]. However, the hallmark of brain neuroinflammation is microglia activation, which releases highly proinflammatory cytokines, ROS, and NO and leads to protein oxidation, lipid peroxidation, DNA fragmentation, neuronal inflammation, and cell death [78, 134]. Microglial cells are the resident macrophage-like population within the CNS and are a prime component of the brain immune system. In physiological conditions, microglia actively survey the microenvironment and ensure normal CNS activity by secreting neurotrophic factors (i.e., NGF). Although microglial activation plays an important role in the phagocytosis of dead cells in the CNS, overactivated microglia cause inflammatory responses that lead to neuronal and axonal degeneration and disruption of the immature BBB [135].

Inflammatory mediators such as interleukin-1*β* (IL-1*β*), interferon-*γ* (IFN-*γ*), tumor necrosis factor-*α* (TNF-*α*), and NO are produced by activated microglia and have recently been linked to the pathogenesis of neurological disorders [136]. Therefore, pharmacological interference with the overactivation of microglia may have a therapeutic benefit in the treatment of inflammation-mediated neurological disorders [137]. The activities of resveratrol against neuroinflammation appear to target activated microglia and result in the reduction of proinflammatory factors (i.e., TNF-*α*, IL-*β*, prostaglandin E2, cyclooxygenases, and iNOS through the modulation of signal transduction pathways) [138].

**Figure 3:**
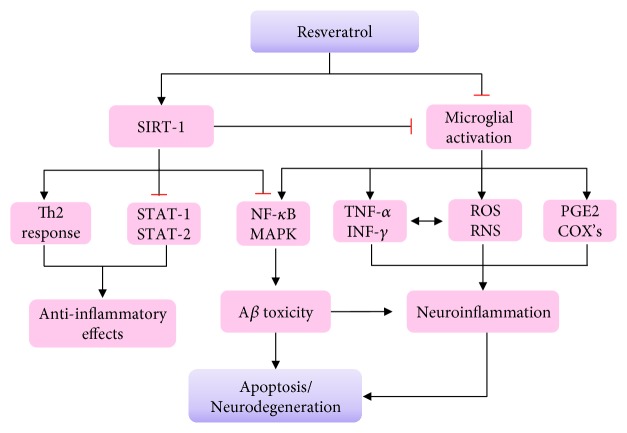
Anti-inflammatory effects of resveratrol and the role of SIRT1 in AD.

Gocmez et al. [139] showed that aging increased the levels of TNF-*α* and led to chronic neuroinflammation in the hippocampus and impaired spatial learning and memory. However, chronic administration of resveratrol reversed the cognitive deficits and inhibited the production of inflammatory cytokines. In addition, resveratrol also inhibited the activation of signal transducer and activator of transcription (STAT1 and STAT3) and prevented the proinflammatory effect of A*β* and A*β*-triggered microglial activation [140]. However, the role of resveratrol in microglia activation and the molecular mechanisms involved are not fully elucidated. The major pathway seems to involve SIRT1 activation, which promotes Th2 responses by increasing anti-inflammatory cytokine expression and upregulating PGC-1*α* (Figure 3) [141, 142].

#### 3.2.1. In Vitro Studies

Resveratrol has numerous functions in neuroinflammation, as it induces mitophagy [143, 144]. Wang et al. [80] used a differentiated lineage of cell lymphomas from rat pheochromocytoma as a cellular model of AD treated with A*β* peptide A*β*1–42 (A*β*1–42). Resveratrol decreased the mitophagy-mediated mitochondrial damage and attenuated the oxidative stress caused by A*β*1–42 [141]. Neuroinflammation may also be related to the degradation of the BBB [145]. The BBB is constituted of structural and functional elements such as brain endothelial cells [146, 147]. Thus, Annabi et al. [145] demonstrated that human brain microvascular endothelial cells treated with a carcinogen can signal through NF-*κ*B, allowing release of inflammatory markers such as matrix metalloproteinase 9 (MMP-9) and COX-2. However, resveratrol decreased secretion of MMP-9 and expression of COX-2 [145]. It also activated the expression of SIRT1, which regulated inflammation, inhibited NF-*κ*B signaling, and prevented A*β*-induced degeneration [148].

#### 3.2.2. Animal Studies

Several studies suggest that pharmacological activation of SIRT1 may represent a promising approach to prevent amyloid deposition and neurodegeneration in AD [99, 149]. The relationship between SIRT1 and AD is paramount, as a study of the SIRT1 serum concentration in healthy subjects and AD patients showed a reduced serum SIRT1 concentration that correlated with the increasing age of an individual. The decline was much more pronounced in patients with AD [93].

SIRT1 also exhibited therapeutic activity in a transgenic mouse model of AD [150]. Wang et al. [127] assessed an alternative therapy for AD that used mesenchymal stem cells derived from the umbilical cord combined with resveratrol in a mouse model of AD. Resveratrol also favored the formation of neurons and regulated SIRT1 expression in the hippocampus of AD rats [127]. Resveratrol has anti-inflammatory functions and can inhibit A*β*-induced NF-*κ*B signaling in microglia and astrocytes [151]. Another study showed that mice overexpressing SIRT1 exhibited reduced brain inflammation (due to its action in tau phosphorylation) and reduced cognitive defects that were specific to the APP transgenic mouse [149, 150].

#### 3.2.3. Human Studies

Some neurodegenerative diseases, such as AD, are associated with oxidative stress and neuroinflammation, and proteins that are closely related to this neurological disorder (i.e., AMPK, SIRT1, and PGC-1*α*) can be modulated by resveratrol [152]; however, there are few clinical studies on resveratrol in AD patients. Moussa et al. [153] reported that patients treated with resveratrol (1 g/day) for 52 weeks demonstrated reduced MMP-9 levels (an inflammatory marker related to AD) compared to a placebo group. In addition, patients treated with resveratrol had less cerebrospinal fluid decline, which resulted in less A*β* accumulation in the brain. Resveratrol probably strengthened the CNS, hampered the penetration of MMP-9, and reduced the activity of this inflammatory agent [154].

The anti-inflammatory effects of resveratrol are mediated, at least in part, by suppressing the activation of NF-*κ*B, extracellular signal-regulated kinase-1 and kinase-2, and mitogen-activated protein kinase (MAPK) signaling pathways, which are all important upstream modulators of the production of proinflammatory mediators [137]. Resveratrol-mediated overexpression of SIRT1 markedly reduced NF-*κ*B signaling and A*β*-mediated microglial activation and had strong neuroprotective effects [68, 155]. The polymerization of A*β* peptides was markedly inhibited by resveratrol, which stimulated the proteasomal degradation of A*β* peptides [30, 75].

Studies strongly suggest that resveratrol-induced SIRT1 inhibits NF-*κ*B signaling in microglia and astrocytes and protects AD neurons against A*β*-induced toxicity. This NF-*κ*B signaling controls the expression of iNOS, which mediates apoptosis and neurodegeneration [32]. Resveratrol also effectively suppresses the apoptotic activities of both p53 and FOXO via SIRT1 overexpression and confers neuronal protection in AD [152, 156].

Therefore, the potential anti-inflammatory mechanisms for resveratrol-mediated neuroprotection involve (i) reduction of proinflammatory cytokine expression, (ii) suppression of MAPK signal transduction pathways, and (iii) activation of the SIRT1 pathway, which in turn suppresses the activation of the NF-*κ*B signaling pathway and protects neurons against microglia-dependent A*β* toxicity [134].

In this context, the neuroprotective effects of resveratrol can involve the scavenging of ROS, decreased NO levels, improved antioxidant capacity, NF-*κ*B inhibition, inhibition of inflammatory mediators, promotion of neuronal survival via SIRT1 activation [157, 158], the prevention of DNA lesions, and the prevention of lipid peroxidation in cell membranes [85]. Animal models also indicate that resveratrol improves the spatial memory by decreasing the accumulation of A*β* peptides and lipid peroxidation in the hippocampus, thus protecting against neuronal apoptosis [159].

Therefore, it is also important to emphasize that these neuroprotective effects can also be mediated by other action mechanisms of resveratrol. Another neuroprotective mechanisms of resveratrol include the following: (i) inhibits the tauopathy by interfering with the MID1-PP2A (midline 1-protein phosphatase 2A) complex or by altering or partially inhibiting of the glycogen synthase kinase 3 beta (GSK3*β*) and p53 interaction [6, 110]; (ii) improves learning and long-term memory formation through the microRNA (microribonucleic acid)-CREB (cAMP response element-binding protein)-BDNF pathway [20]; (iii) protects against A*β*-mediated neuronal impairment (inflammation and oxidative stress) by activation of AMP-activated protein kinase- (AMPK-) dependent signaling and inhibition of NF-*κ*B expression and iNOS levels [160]; (iv) antioxidative activity by reduction in levels of ROS enhances the expression of various antioxidant defensive enzymes (heme oxygenase 1, catalase, glutathione peroxidase, and superoxide dismutase), downregulation of prooxidative stress proteins (i.e., plaque-induced glycogen synthase kinase-3*β* (GSK-3*β*), and AMPK [8, 10]; (v) improves cognitive impairment due to inhibition of cholinesterase activity [161]; (vi) inhibits the A*β* plaque synthesis by restoration of normal cellular autophagy via the TyrRS-PARP1 (auto-poly-ADP-ribosylation of poly (ADP-ribose) polymerase 1)-SIRT1 signaling pathway and enhancement of transthyretin (transporter protein) binding to A*β* oligomers [162]; (vii) inhibits mammalian target of rapamycin (mTOR) signaling and induces AMPK, thereby stimulating the clearance of A*β* aggregates [110]; (viii) prevents the neuronal cell death by attenuating apoptosis via Akt/p38 MAPK signaling and inhibits caspase-3 and B cell lymphoma-2 (Bcl-2)/Bcl-2-associated X protein signaling [163, 164]; (ix) increases intracellular calcium levels, promoting the activation of calcium/calmodulin-dependent protein kinase kinase *β*-CamKK*β*-AMPK pathway, which alters mitochondrial function and leads to a decrease in ROS generation [165]; (x) attenuated injury and promoted proliferation of the neural stem cells, at least in part, by upregulating the expression of nuclear factor (erythroid-derived 2)-like 2 (Nrf2), HO-1, and NAD(P)H:quinone oxidoreductase 1 (NQO1) [166]; and (xi) inhibits the neuronal electrical activity by mechanisms associated with large conductance of Ca^2+^ potassium channels and attenuates A*β*-induced early hippocampal neuron excitability impairment [167]. Therefore, resveratrol may be an important tool to protect neuronal cells from oxidative damage and a promising strategy in the treatment of AD.

## 4. Conclusions

Resveratrol is a potential compound for the treatment of AD due to its antioxidant and anti-inflammatory properties. The key neuroprotective mechanism of resveratrol in AD seems to be linked with SIRT1 activation. Although the mechanisms that link resveratrol to the overexpression of SIRT1 and neuroprotection are unknown, this expression may play an important role in neuronal protection from ROS, NF-*κ*B signaling in activated microglia, prevent A*β* toxicity, and contribute to improved learning and memory function. Resveratrol can also effectively suppress the apoptotic activities of both p53 and FOXO via SIRT1 overexpression and confer neuronal protection in AD. Although this review focuses on the importance of SIRT1 activation for the neuroprotective role of resveratrol, it is also important to clarify that these mechanisms are still unclear and fully elucidated. In addition, resveratrol may act on CNS by inhibiting neuroinflammatory and prooxidant mechanisms by multiple action mechanisms that are independent of SIRT-1. These mechanisms are quite complex and involve stimulation or inhibition of multiple signaling pathways or alteration of potassium channels eading to inhibition of neuronal electrical activity. In summary, the major mechanisms that may be associated with the neuroprotective effect of resveratrol, in addition to SIRT1, include stimulation of regulation by microRNA-CREB-BDNF pathway, inhibition of mTOR and AMPK-dependent signaling pathways, inhibition of enzymes (cholinesterase activity), transcription factor (NF-*κ*B) and apoptotic pathways, and stimulation of cellular autophagy and expression of Nrf2, HO-1, NQO1, among others. Therefore, we critically analyze and suggest that SIRT1 is one of the main mechanisms related to the beneficial effects of resveratrol; however, this compound can change multiple pathways simultaneously, and then, there is a need for crosstalk between signaling and regulatory functions to provide improvements in the development and progression of AD. In addition, caution is required in therapies with natural products, since intrinsic aspects of the patient, environmental factors, and characteristics of the compound studied are important for efficacy and therapeutic success.

Despite the neuroprotective potential of resveratrol demonstrated in several in vitro studies, the major limitation currently facing is the lack of information from clinical studies that correlates the SIRT1 activation and the inflammatory and oxidative status reduction associated with improvement in the development and progression of AD. Overall, evidence from clinical trials is weak and largely inconclusive. Most human studies establish a link between consumption of foods rich in resveratrol and reducing the incidence or prevalence of AD, as well as improvement in learning, memory, visual and spatial orientation, and social behavior. However, these observed effects may be the result of complex direct and indirect interactions of the various constituents present in the diet, not only of resveratrol. In addition, other difficulties in clinical trials are the following: (i) the studies are mainly conducted with volunteers, not reflecting the target population, (ii) the participants' age is quite broad between 18 and over 80 years of age, and (iii) sample size is rarely calculated and the slow progression of AD is not investigated because it requires longer clinical time in the trials. Another important issue is the poor bioavailability of resveratrol, which makes it difficult to link with the optimal concentrations achieved in in vitro experiments. Although preclinical studies also indicate that resveratrol is able to cross the blood-brain barrier, low concentrations of this molecule have been detected in the brain, and only higher concentrations of resveratrol and its metabolites have been found in the blood. In addition, it is emphasized that the neuroprotective effects of resveratrol are mainly short term, varying according to dose, dosage form, duration of treatment, pharmacokinetic and pharmacogenetic parameters, food and drug interactions, among others. Thus, we conclude that, to date, evidence based on clinical studies is still insufficient, contradictory, and inconclusive, so we recommend that further clinical trials be conducted to substantiate the neuroprotective effects of resveratrol and its likely mechanisms of action in the body. However, we emphasize that resveratrol is promising in health promotion, not only for its antioxidant activities but also for its anti-inflammatory and neuroprotective properties. Thereby, further studies assessing other routes of administration or pharmaceutical formulations (i.e., nanoencapsulation) are required to improve the tissue-targeting concentration and allow resveratrol to exert its biological activities in AD.

## Figures and Tables

**Table 1:** Resveratrol concentration in food sources.

Food source	Family	Resveratrol content	Reference
Banana peel (*Musa* sp.)	Musaceae	38.8 ± 0.1 mg/100 g	[41]
Caper bush (*Capparis spinosa*)	Capparidaceae	235.31 mg/100 g	[42]
Whole grapes (*V. vinifera*)	Vitaceae	8.4 ± 0.2 mg/100 g	[41]
White wine (*V. vinifera* cv. Chardonnay)	Vitaceae	0.04 ± 0.01 mg/l	[43]
Red wine (*V. vinifera* cv. Shiraz)	Vitaceae	0.53 ± 0.06 mg/l	[43]
Mulberry wine (*Morus rubra*)	Moraceae	145.31 ± 8.89 mg/l	[43]
Whole Mentha (*Mentha arvensis*)	Lamiaceae	9.4 ± 0.0 mg/100 g	[41]
Boiled peanuts (*Arachis hypogaea*)	Fabaceae	5.1 ± 2.8 *μ*g/g	[40]
Peanut butter (*A. hypogaea*)	Fabaceae	0.3 ± 0.1 *μ*g/g	[40]
Pomegranate pulp (*Punica granatum*)	Punicaceae	19.9 ± 0.2 mg/100 g	[41]
Whole spinach (*Spinacia oleracea*)	Amaranthaceae	19.3 ± 0.1 mg/100 g	[41]
